# Cardiovascular Mortality Risk in Patients with Bladder Cancer: A Population-Based Study

**DOI:** 10.3390/jcdd9080255

**Published:** 2022-08-09

**Authors:** Shunde Wang, Chengguo Ge, Junyong Zhang

**Affiliations:** 1 Department of Urology, The ChenJiaqiao Hospital of ShaPingba District of Chongqing City, Chongqing 401331, China; 2 Department of Urology, The Second Affiliated Hospital of Chongqing Medical University, Chongqing 400010, China

**Keywords:** bladder cancer, cardiovascular mortality, SEER, competing risk regression

## Abstract

Background: The purpose of this study was to evaluate the risk of cardiovascular mortality (CVM) among patients with bladder cancer (BC). Methods and Materials: Data were collected from the Surveillance, Epidemiology, and End Results (SEER) database for patients who were diagnosed with BC by pathology between 2000 and 2016. The standardized mortality rate (SMR) was calculated based on reference data from the general population. Nelson–Aalen cumulative hazard curves were used to assess the risk of experiencing CVM in BC patients. Multivariate competing risk models were performed. Results: In total, data from 237,563 BC patients were obtained from the SEER database for further analysis, of which 21,822 patients experienced CVM; the overall SMR for CVM in BC patients was 1.16 (95% CI: 1.14–1.17). Age, race, sex, year of diagnosis, histologic type, summary stage, surgery, marital status, and college education level were independent predictors of CVM in patients with BC. Conclusions: Patients with BC have a significantly increased risk of experiencing CVM compared to the general population. Pre-identification of high-risk groups and cardiovascular protection interventions are important measures to effectively improve survival in this group of patients.

## 1. Introduction

It is estimated that there are more than 500,000 new cases of bladder cancer (BC) worldwide each year, with approximately 40% of these resulting in death. However, the US alone accounts for 16% of all new cases worldwide each year [1,2,3].

Cardiovascular diseases (CVDs) are one of the primary causes of death worldwide. According to a study published in The Lancet in 2018, cardiovascular mortality (CVM) increased by 2.1% over a 10-year period from 2007 [4]. A total of 17.9 million people died from CVD in 2019, contributing to 32% of all deaths worldwide, while only about 10 million people died from cancer in 2019. In 2019, Kochanek et al. reported that in the US, over 0.64 million deaths were due to heart disease, while nearly 0.6 million deaths were attributed to primary cancers [5].

With the improvement in the quality of life and medical care, patients’ life expectancy has increased, and mortality from primary cancers has gradually decreased, leading to the prominence of mortality factors for non-primary cancers, among which CVD is one of the leading causes of death for non-cancers [6]. Several published studies have indicated that cancer patients are at significantly higher risk of experiencing CVM than the general population for a variety of reasons [7,8,9].

A detailed literature search revealed no reports of CVM in patients with BC. Therefore, our discoveries may help to establish a more targeted follow-up strategy for BC patients as well as more effective CVM prevention measures.

## 2. Methods

### 2.1. Data Source and Patient Selection

**Figure 1:**
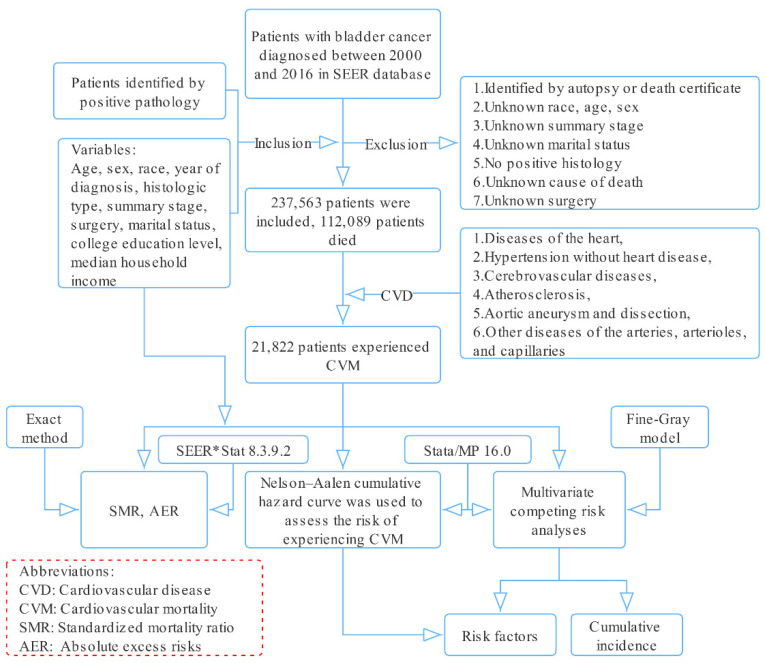
Selection of eligible patients and study design.

Information related to patients with BC diagnosed from 2000 to 2016 was downloaded from the Surveillance, Epidemiology, and End Results (SEER) database using SEER*Stat software (National Cancer Institute, Bethesda, MD, USA, version 8.3.9.2, Database: Incidence—SEER 18 Regs excluding AK Research Data, November 2018 Sub (2000–2016) for standardized mortality ratios (SMRs)) (Figure 1).

Patients diagnosed with BC with positive pathology from 2000 to 2016 were included. The following histological codes were used: 8000, 8004, 8010, 8012, 8013, 8020–8022, 8030–8033, 8041, 8042, 8045, 8046, 8050–8052, 8070–8075, 8082, 8083, 8120–8122, 8130, 8131, 8240, 8244, 8246, 8490, 8507, 8542, 8560, 8570, 8574–8576, 8940, and 8980 (International Classification of Diseases for Oncology, 3rd edition).

Patients identified only by autopsy or death certificate and patients with incomplete data for certain variables (age, sex, race, etc.) were excluded.

CVM was the primary endpoint of interest, defined by the following six CVDs in the SEER database: (1) diseases of the heart, (2) hypertension without heart disease, (3) cerebrovascular diseases, (4) atherosclerosis, (5) aortic aneurysm and dissection, (6) other diseases of the arteries, arterioles, and capillaries [10,11], while competing events were deaths from BC, other cancers, and other non-cancer diseases.

### 2.2. Study Variables

The definitions and information regarding variables are as follows: age at diagnosis (0–50, 51–60, 61–70, 71–80, 81+), race (White, Black, American Indian/Alaska Native, Asian or Pacific Islander), sex (male, female), year of diagnosis, histologic type (Tcc: transitional cell carcinoma; Scc: squamous cell carcinoma; Ac: adenocarcinoma; Nec: neuroendocrine carcinoma; Oet: other epithelial tumors), summary stage (in situ, localized, regional, distant), surgery (no surgery; TURBT: transurethral resection of bladder tumor; PC: partial cystectomy; RC: radical cystectomy), marital status (married, separated, divorced, widowed, unmarried (unmarried or domestic partner, single)), college education level, median household income, cause of death, and follow-up time.

### 2.3. Statistical Analysis

The SMR is the ratio of the number of observed deaths to the number of expected deaths of CVM [12]. We used an exact method to calculate the 95% confidence intervals (95% CIs) for all SMRs. Absolute excess risks (AERs) were also calculated, which are a proxy for the excess number of deaths per 10,000 person-years in different subgroups [10,12]. The Nelson–Aalen cumulative hazard curve was used to assess the risk of experiencing CVM in different subgroups of BC patients. Multivariate competing risk analyses were conducted to identify risk factors associated with CVM [13].

All analyses were conducted using SEER*Stat software (version 8.3.9.2, National Cancer Institute, Bethesda, MD, USA), Stata/MP version 16.0 (Stata Corp, College Station, TX, USA), and Microsoft Excel 2019 (Microsoft, Redmond, WA 98052-6399, USA). A two-sided *p*-value < 0.05 was considered statistically significant.

## 3. Results

### 3.1. Patient Characteristics

For this study, a total of 237,563 patients diagnosed with BC were identified from 2000 to 2016. The mean age was 70.82 ± 12.04 years, and the median follow-up time was 49 months. Most of the patients were over 71 years old (54.93%), White (90.40%), male (75.72%), married (64.58%), and had carcinoma in situ (52.79%). The histologic types of BC consisted of Tcc (95.63%), Scc (1.59%), Ac (0.97%), Nec (0.65%), and Oet (1.15%). A total of 201,433 (84.79%) patients underwent TURBT, 3,278 (1.38%) patients underwent PC, 20,999 (8.84%) patients underwent RC, and 11,853 (4.99%) patients did not undergo surgery. The largest number of deaths occurred during the follow-up period of <1 year (32.81%), followed by the 1- to 3-year follow-up period (28.07%). Among the 112,089 patients that died during the follow-up period, 21,822 patients experienced CVM, with the main cause being disease of the heart (70.14%), followed by cerebrovascular disease (12.31%), hypertension without heart disease (2.89%), atherosclerosis (0.97%), aortic aneurysm and dissection (1.14%), and other diseases of the arteries, arterioles, and capillaries (1.06%).

**Figure 2:**
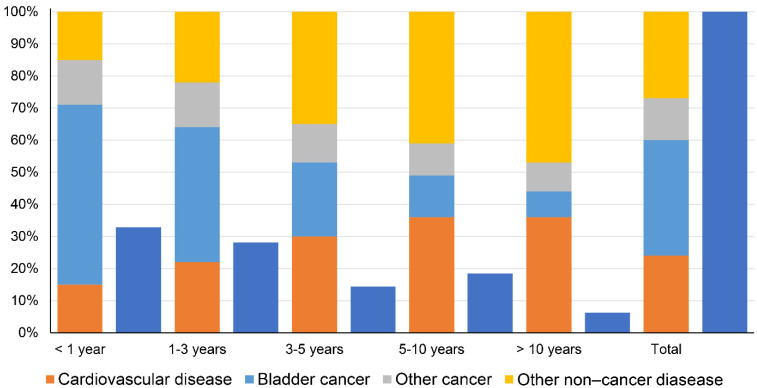
Causes of death in each latency period following bladder cancer diagnosis (dark blue indicates the percentage of the total number of deaths for each latency period).

A total of 37,761 BC patients died within 1 year after diagnosis, including 20,954 (55.49%) deaths from BC, 5660 (14.99%) deaths from CVD, 5381 (14.25%) deaths from other cancers, and 5766 (15.27%) deaths from other non-cancer diseases. The proportion of cancer-related deaths decreased gradually at <1 year, 1–3 years, 3–5 years, 5–10 years, and >10 years (including BC and other cancers), while the proportion of non-cancer disease-related deaths increased gradually (including CVD and other non-cancer diseases) (Figure 2).

### 3.2. SMR and AER

The overall SMR for CVM was 1.16 (95% CI: 1.14–1.17), and the AER was 27.18/10,000 person-years in BC patients. Table 1 shows the baseline features and SMRs of CVM in patients with BC.

The SMRs of the six causes of CVM in patients with BC are shown in Table 2. The most significant increase in the SMR was found for aortic aneurysm and dissection (SMR [95% CI]: 1.31 [1.15–1.47]), followed by other diseases of the arteries, arterioles, and capillaries (SMR [95% CI]: 1.20 [1.02–1.38]), atherosclerosis (SMR [95% CI]: 1.18 [1.09–1.28]), diseases of the heart (SMR [95% CI]: 1.16 [1.13–1.19]), cerebrovascular diseases (SMR [95% CI]: 1.13 [1.06–1.24]), and hypertension without heart disease (SMR [95% CI]: 1.05 [1.01–1.09]).

**Figure 3:**
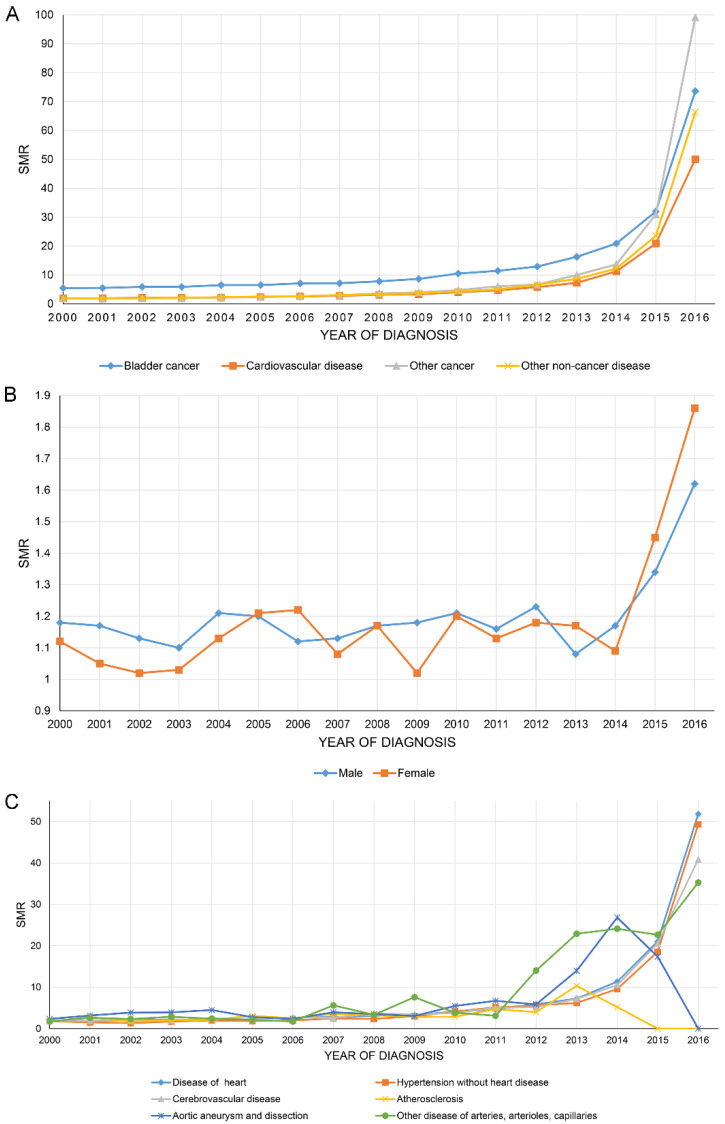
The overall standardized mortality ratio (SMR) of all causes of death of bladder cancer patients increased year by year (**A**). SMR in cardiovascular disease stratified by sex (**B**) and all causes of cardiovascular mortality (**C**).

Figure 3A shows that the SMR of all causes of death of BC patients increased year by year, with BC having the highest SMR of all. Figure 3B,C show the SMR in CVD stratified by sex and all causes of CVM.

**Figure 4:**
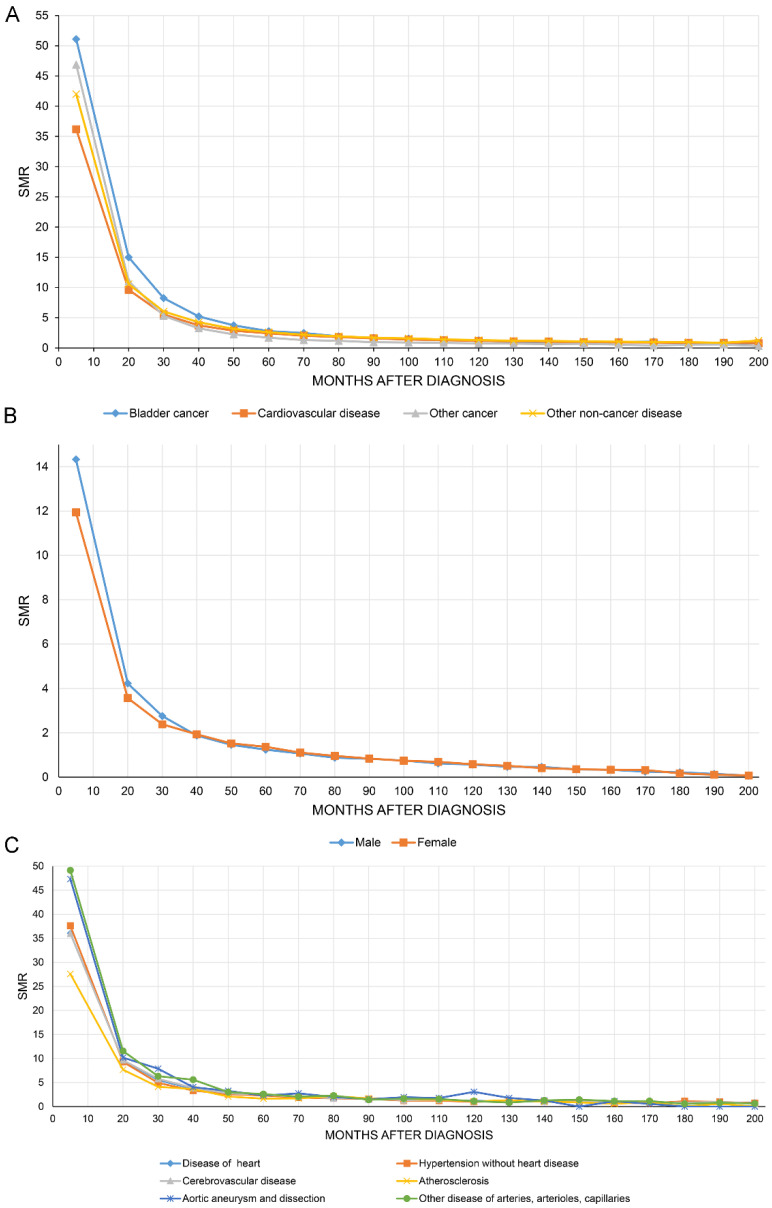
The overall standardized mortality ratio (SMR) of all causes of death in bladder cancer patients decreased with increasing follow-up time (**A**). SMR in cardiovascular disease stratified by sex (**B**) and all causes of cardiovascular mortality (**C**).

Figure 4A shows that the SMR of all causes of death of BC patients decreased with increasing follow-up time. Figure 4B,C show the SMR for CVD stratified by sex and all causes of CVM.

### 3.3. Nelson–Aalen Cumulative Hazard Curve

**Figure 5:**
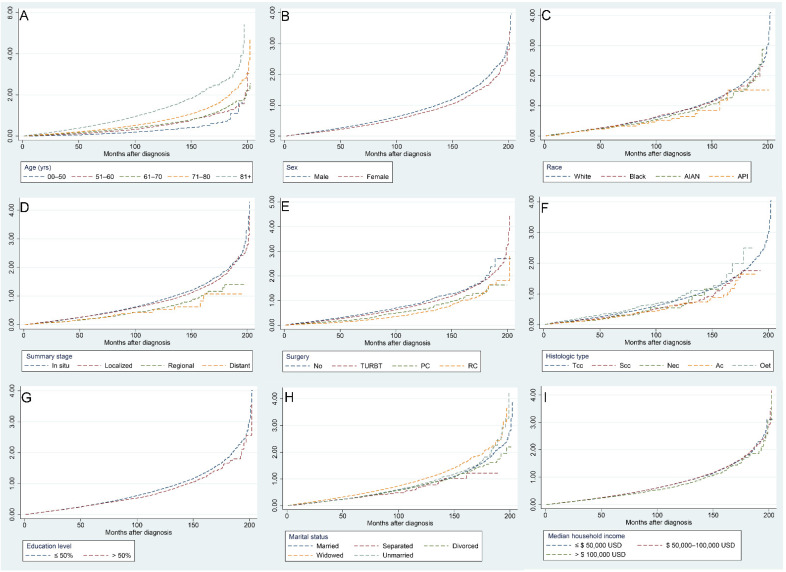
Independent Nelson–Aalen cumulative hazard curves for various factors of cardiovascular mortality in bladder cancer patients: age (**A**), sex (**B**), race (**C**), summary stage (**D**), surgery (**E**), histologic type (**F**), education level (**G**), marital status (**H**), and median household income (**I**).

Figure 5 illustrates the risk of CVM with increasing follow-up time for different factors, with the following subgroups associated with a higher risk of CVM: age over 71 years, male sex, White, carcinoma in situ and localized tumors, no surgery, Tcc and Nec pathological types, a college education level less than 50%, widowed, and a median household income less than USD 100,000.

**Figure 6:**
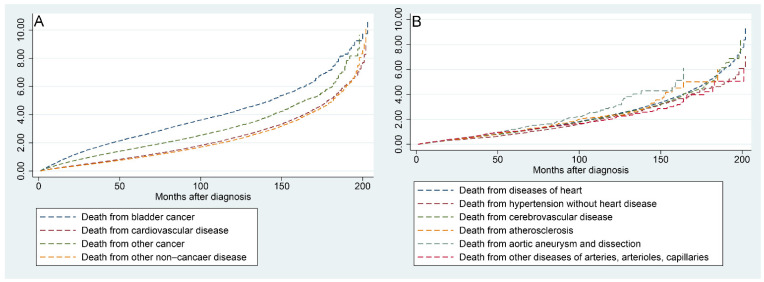
Nelson–Aalen cumulative hazard curves for all causes of death in primary bladder cancer patients (**A**) and stratified by the six factors of cardiovascular disease (**B**).

Figure 6A shows that the risk of all mortality factors in BC patients increased with the time to follow-up. Figure 6B shows that the risk of the six factors contributing to CVM increased progressively with the time to follow-up.

**Figure 7:**
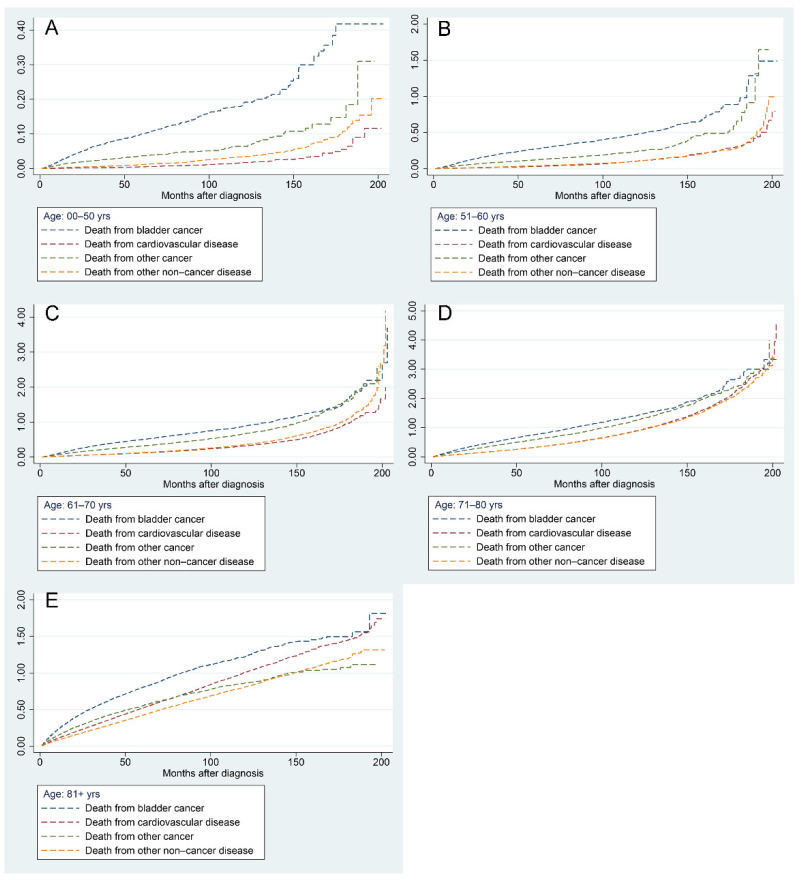
Nelson–Aalen cumulative hazard curves for all causes of death in primary bladder cancer patients in different age groups: 0–50 years (**A**), 51–60 years (**B**), 61–70 years (**C**), 71–80 years (**D**), and 81+ years (**E**).

Figure 7 demonstrates the results of a progressive increase in the risk of all mortality factors in BC patients of different ages with increasing follow-up time. The risk of BC-related death was highest in patients aged less than 70 years, followed by other cancers, other non-cancer diseases, and CVDs (Figure 7A–C). In patients aged 71–80 years, the risk of CVD surpassed that of other non-cancer diseases and ranked third when the follow-up time exceeded 120 months (Figure 7D). In patients older than 81 years, the risk of CVD-related mortality surpassed that of other cancers as the second leading risk factor at approximately 80 months of follow-up (Figure 7E).

### 3.4. Predictors of Death from CVD

**Figure 8:**
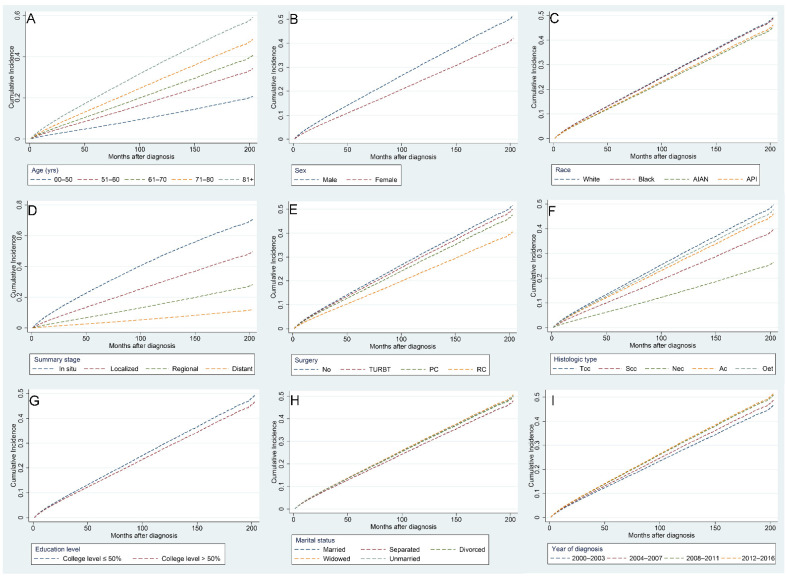
Cumulative incidence curves for various factors of cardiovascular mortality in bladder cancer patients after competing risk regression analysis: age (**A**), sex (**B**), race (**C**), summary stage (**D**), surgery (**E**), histologic type (**F**), education level (**G**), marital status (**H**), and year of diagnosis (**I**).

Risk factors associated with CVM in BC patients were identified using multivariate competing risk regression analysis (Table 3). We found that the following indicators were independently related to higher risks of CVM: age over 81 years (HR [95% CI]: 3.855 [3.285–4.525]), diagnosed between 2012 and 2016 (HR [95% CI]: 1.149 [1.094–1.208]), widowed (HR [95% CI]: 1.079 [1.043–1.116]), and unmarried (HR [95% CI]: 1.066 [1.016–1.119]). On the contrary, the following indicators were found to be independently related to lower risks of CVM: American Indian/Alaska Native (HR [95% CI]: 0.895 [0.827–0.968]), female (HR [95% CI]: 0.755 [0.73–0.78]), Scc histological type (HR [95% CI]: 0.446 [0.349–0.571]), distant summary stage (HR [95% CI]: 0.105 [0.093–0.119]), undergoing RC (HR [95% CI]: 0.715 [0.652–0.784]), and a college education level >50% (HR [95% CI]: 0.926 [0.87–0.986]). Figure 8 shows the CIF curves using Fine–Gray competing risk analyses.

## 4. Discussion

In this large population study based on the SEER database, we analyzed the long-term CVM of patients with BC. Although the number of diagnoses of BC is increasing every year, the number of people who develop CVM is decreasing. This may be related to the advancement of BC treatment strategies and the improved quality of comprehensive cancer management. At the same time, the treatment of CVD and the ability to cope with cardiovascular events have also improved, which has effectively reduced the incidence of CVM.

Published studies have illustrated that the risk of CVM varies considerably between patients with cancer at different primary sites [14,15,16]. In this study, we focused only on CVM in patients with BC. By studying 21,822 patients, we found that the risk of CVM in patients with BC was approximately 16% higher than that in the US general population (SMR [95% CI]: 1.16 [1.14–1.17]). Over the entire follow-up period, patients with BC had an increased risk of CVM from all causes. Our study identified age, race, sex, year of diagnosis, histologic type, summary stage, surgery, marital status, college education level, and median household income as independent predictors for the development of CVM in patients with BC.

Similar to the results previously published by Zaorsky et al. [17], we found that the risk of CVM in BC patients was highest in the first 10 months after diagnosis. Meanwhile, the study by Fang and Ye et al. suggested that newly diagnosed cancer may lead to psychological and emotional distress in cancer patients, which in part promotes the development of CVM [18,19]. Therefore, psychiatric assessment and psychological support are necessary for newly diagnosed BC patients. Using the Nelson–Aalen hazard curves, we found that the risk of CVM in BC patients gradually increased with increasing age at diagnosis. Primary cancer is the most common cause of death for most cancer patients. However, our study found that the risk of CVM ranked first when cancer patients were diagnosed at an age of >71 years. These results suggest that clinicians should focus not only on BC itself, but also on the risk factors for experiencing CVM in patients of advanced age.

Multivariate competing risk regression analysis was used to identify risk factors associated with CVM in BC patients. We also observed that patients with BC aged over 81 years had the highest CVM (HR [95% CI]: 3.855 [3.285–4.525]) and a lower SMR (SMR [95% CI]: 1.18 [1.15–1.20]), but patients aged 51–60 years had the highest SMR (SMR [95% CI]: 1.34 [1.25–1.43]), which is similar to the findings of Zaorsky et al. [17]. Men have a higher risk of CVM, which may be related to smoking, alcohol consumption, or higher work stress, all of which are independent risk factors for CVD [20,21,22,23]. Additionally, our study demonstrated that unmarried (HR [95% CI]: 1.066 [1.016–1.119]) and widowed (HR [95% CI]: 1.079 [1.043–1.116]) BC patients are at a higher risk of CVM, which may be associated with the fact that married patients are more likely to receive encouragement and support from their spouses, both emotionally and physically [24]. Additionally, some studies pointed out that marriage helps improve cardiovascular, endocrine, and immune function as well as cancer prognosis [25,26]. Patients with a lower socioeconomic status have been reported to be at higher risk for CVM [12,27], and our findings demonstrate that patients with lower levels of college education had a higher risk of CVM, in accordance with previous results.

In this study, the majority of patients underwent surgery (94.98%), including TURBT (84.71%), PC (1.41%), and RC (8.86%). Although only 1161 (5.30%) patients in this study did not receive surgery, the SMR (SMR [95% CI]:1.49 [1.40–1.57]) was the highest. Multivariate competing risk analysis showed that patients with BC who underwent RC had the lowest risk of developing CVM (HR [95% CI]: 0.715 [0.652–0.784]). This is probably explained by the fact that most patients who underwent RC surgery had an advanced tumor stage and did not have enough life expectancy to experience a CVM event (median survival time: 28 months for TURBT, 20 months for PC, 16 months for RC, and 11 months for no surgery). Our results show that patients with BC who are not treated with surgery have the highest risk of CVM, although the median survival time was only 11 months. One possible reason is that the diagnosis of BC often causes a longer period of psychological and emotional distress, and untreated BC often progresses rapidly, which would result in patients being at a higher risk of experiencing CVM [28,29].

Many risk factors are shared between cancer and CVD, such as smoking, radiation, air pollution, and metabolic syndrome [30]. Recent studies have shown that there is also a direct interplay between cancer and CVD, with anthracyclines having significant cardiotoxic effects that can lead to CVD such as heart failure or atherosclerosis during or years after anticancer treatment [31,32,33]. In addition to risk factors, the genetic background plays an important role in the interaction between cancer and CVD [30]. Studies have shown that mutations in age-related clonal hematopoiesis of indeterminate potential accelerate the development of CVD such as atherosclerosis or coronary artery disease [34,35,36], further worsening the prognosis of patients with heart failure [37]. Recent studies have demonstrated that heart failure can promote the transition to the pre-tumor stage and tumor growth [38,39,40]. In cancer patients, there is a high incidence of cardiomyopathy-related mutations, such as the DNA damage response/repair system, and mutations in the DNA damage response/repair system gene increase the risk of cardiotoxicity with anticancer therapy [41]. Meanwhile, pathophysiological alterations in hereditary cardiomyopathy can promote cancer development and progression and may further increase the cardiotoxic effects of anticancer therapies [30,42,43].

There are still some shortcomings in our study. First, information related to CVD, such as smoking, alcohol consumption, and the presence of congenital diseases, was not recorded in the SEER database. Second, there was no further analysis of the effects of chemotherapy, radiotherapy, and some other new therapeutic strategies on CVM. Meanwhile, some studies reported that the causes of CVM on death certificates might have been overestimated [44], which might have affected the accuracy of our study to some extent.

## 5. Conclusions

In summary, patients with BC have a significantly increased risk of developing CVM compared to the general population. This suggests that early screening for CVD and the assessment and monitoring of risk factors for CVM should be performed after the diagnosis of BC. It also provides important guidance on how BC patients should be followed up and educated about their associated health risks. In addition, further investigations are needed to understand the mechanisms by which BC patients develop CVD, and to design effective prevention and monitoring strategies.

## Figures and Tables

**Table 1:** Baseline features and standardized mortality ratios of cardiovascular mortality in patients with bladder cancer.

	Observed Deaths	Expected Deaths	SMR [95% CI]	AER per 10,000	Persons	Person-Years at Risk
Total	21,822	18,888.75	1.16 # [1.14–1.17]	27.18	237,563	1,079,006.59
Age (yrs)						
00–50	147	120.54	1.22 # [1.03–1.43]	2.86	13,009	92,394.95
51–60	898	670.57	1.34 # [1.25–1.43]	11.57	32,856	196,551.81
61–70	2921	2378.56	1.23 # [1.18–1.27]	17.57	61,201	308,691.31
71–80	7874	7244.13	1.09 # [1.06–1.11]	19.51	75,277	322,772.51
81+	9982	8474.95	1.18 # [1.15–1.20]	95.02	55,220	158,596.01
Race						
White	20,093	17,534.79	1.15 # [1.13–1.16]	25.98	214,764	984,523.07
Black	1064	849.60	1.25 # [1.18–1.33]	42.24	13,045	50,763.83
AIAN	39	17.98	2.17 # [1.54–2.97]	86.85	619	2420.49
API	626	486.39	1.29 # [1.19–1.39]	33.81	9135	41,299.21
Sex						
Male	17,080	14,647.53	1.17 # [1.15–1.18]	30.00	179,880	810,942.96
Female	4742	4241.22	1.12 # [1.09–1.15]	18.68	57,683	268,063.63
Year of diagnosis						
2000–2003	8044	7142.72	1.13 # [1.10–1.15]	24.01	53,447	375,401.43
2004–2007	6745	5791.75	1.16 # [1.14–1.19]	28.78	56,320	331,202.90
2008–2011	4649	3975.76	1.17 # [1.14–1.20]	27.50	57,139	244,822.26
2012–2016	2384	1978.52	1.20 # [1.16–1.25]	31.78	706,57	127,580.00
Histologic Type						
Tcc	21,070	18,386.59	1.15 # [1.13–1.16]	25.54	226,953	1,047,766.11
Scc	293	201.34	1.45 # [1.28–1.62]	74.55	3926	12,026.36
Nec	66	44.71	1.48 # [1.14–1.88]	81.80	1565	2602.70
Ac	144	86.84	1.61 # [1.36–1.90]	74.51	2388	7134.40
Oet	249	161.48	1.54 # [1.35–1.74]	105.00	2731	8240.26
Summary stage						
In situ	11,859	11,497.53	1.03 # [1.01–1.05]	5.35	125,403	675,257.36
Localized	8744	6679.56	1.31 # [1.28–1.34]	58.19	86,178	354,786.32
Regional	945	608.73	1.55 # [1.45–1.65]	81.60	17,170	41,207.66
Distant	274	102.93	2.66 # [2.36–3.00]	220.58	8812	7755.25
Surgery						
No	1161	781.75	1.49 # [1.40–1.57]	87.01	11,915	43,585.52
TURBT	19,521	17,082.51	1.14 # [1.13–1.16]	25.53	201,251	955,166.63
PC	236	226.84	1.04 [0.91–1.18]	6.81	3340	13,451.45
RC	904	797.66	1.13 # [1.06–1.21]	15.92	21,057	66,803.00
Marital status						
Married	12,906	12,352.70	1.04 # [1.03–1.06]	7.43	153,408	744,788.58
Separated	109	83.52	1.31 # [1.07–1.57]	38.29	1589	6654.38
Divorced	1431	936.75	1.53 # [1.45–1.61]	59.77	19,239	82,686.26
Widowed	5566	4254.34	1.31 # [1.27–1.34]	99.24	37,708	132,164.93
Unmarried	1810	1261.44	1.43 # [1.37–1.50]	48.67	25,619	112,712.43
Education level						
College level ≦ 50%	20,343	17,373.84	1.17 # [1.15–1.19]	29.93	219,156	991,670.08
College level > 50%	1479	1514.49	0.98 [0.93–1.03]	−4.06	18,407	87,303.01
Median household income						
USD 0–50,000	4155	3206.62	1.30 # [1.26–1.34]	49.17	44,104	192,881.08
USD 50,000–100,000	16,783	14,742.45	1.14 # [1.12–1.16]	24.49	18,2476	832,950.98
Over USD 100,000	884	939.26	0.94 [0.88–1.01]	−10.4	10,983	53,141.02

Abbreviations: SMR, standardized mortality ratio; CI, confidence interval; AER, absolute excess risk; AIAN, American Indian/Alaska Native; API, Asian or Pacific Islander; Tcc, transitional cell carcinoma; Scc, squamous cell carcinoma; Nec, neuroendocrine carcinoma; Ac, adenocarcinoma; Oet, other epithelial tumors; TURBT, transurethral resection of bladder tumor; PC, partial cystectomy; RC, radical cystectomy. # *p* < 0.05.

**Table 2:** The standardized mortality ratios of all causes of cardiovascular mortality in patients with bladder cancer.

CVD	Observed Deaths	Expected Deaths	SMR [95% CI]
Total	21,822	18,888.75	1.16 # [1.14–1.17]
Diseases of the heart	17,293	14,900.46	1.16 # [1.13–1.19]
Hypertension without heart disease	712	678.07	1.05 # [1.01–1.09]
Cerebrovascular diseases	3036	2675.16	1.13 # [1.06–1.24]
Atherosclerosis	239	203.37	1.18 # [1.09–1.28]
Aortic aneurysm and dissection	281	214.32	1.31 # [1.15–1.47]
Other diseases of the arteries, arterioles, and capillaries	261	217.37	1.20 # [1.02–1.38]

Abbreviations: CVD, cardiovascular disease; SMR, standardized mortality ratio. # *p* < 0.05.

**Table 3:** Competing risk regression analysis for predictors of cardiovascular mortality in patients with bladder cancer.

Characteristics	Univariate Analysis		Multivariate Analysis	
	Adjusted HR [95%CI]	p *	Adjusted HR [95%CI]	p *
Age (yrs)				
00–50	Ref.		Ref.	
51–60	2.036 [1.715–2.418]	<0.001	1.804 [1.523–2.137]	<0.001
61–70	2.937 [2.495–3.459]	<0.001	2.249 [1.913–2.644]	<0.001
71–80	4.223 [3.596–4.960]	<0.001	2.858 [2.436–3.354]	<0.001
81+	5.788 [4.929–6.796]	<0.001	3.855 [3.285–4.525]	<0.001
Race				
White	Ref.		Ref.	
Black	0.765 [0.720–0.814]	<0.001	0.989 [0.929–1.052]	0.723
AIAN	0.848 [0.784–0.917]	<0.001	0.895 [0.827–0.968]	0.006
API	0.696 [0.511–0.946]	0.021	0.916 [0.682–1.232]	0.563
Sex				
Male	Ref.		Ref.	
Female	0.739 [0.716–0.763]	<0.001	0.755 [0.730–0.780]	<0.001
Year of diagnosis				
2000–2003	Ref.		Ref.	
2004–2007	1.044 [1.013–1.077]	0.006	1.062 [1.030–1.095]	<0.001
2008–2011	1.003 [0.968–1.039]	0.886	1.133 [1.092–1.174]	<0.001
2012–2016	0.816 [0.779–0.855]	<0.001	1.149 [1.094–1.208]	<0.001
Histologic Type				
Tcc	Ref.		Ref.	
Scc	0.459 [0.409–0.515]	<0.001	0.734 [0.655–0.823]	<0.001
Nec	0.244 [0.192–0.311]	<0.001	0.446 [0.349–0.571]	<0.001
Ac	0.399 [0.338–0.471]	<0.001	0.896 [0.758–1.059]	0.197
Oet	0.686 [0.604–0.778]	<0.001	0.942 [0.830–1.069]	0.358
Summary stage				
In situ	Ref.		Ref.	
Localized	0.553 [0.538–0.568]	<0.001	0.562 [0.546–0.579]	<0.001
Regional	0.204 [0.190–0.218]	<0.001	0.271 [0.251–0.292]	<0.001
Distant	0.087 [0.077–0.098]	<0.001	0.105 [0.093–0.119]	<0.001
Surgery				
No	Ref.		Ref.	
TURBT	1.291 [1.217–1.370]	<0.001	0.953 [0.898–1.011]	0.11
PC	0.665 [0.579–0.764]	<0.001	0.889 [0.773–1.023]	0.099
RC	0.393 [0.361–0.429]	<0.001	0.715 [0.652–0.784]	<0.001
Marital status				
Married	Ref.		Ref.	
Separated	0.730 [0.605–0.879]	0.001	0.999 [0.828–1.205]	0.993
Divorced	0.807 [0.765–0.851]	<0.001	1.055 [0.999–1.113]	0.052
Widowed	1.119 [1.085–1.154]	<0.001	1.079 [1.043–1.116]	<0.001
Unmarried	0.808 [0.770–0.848]	<0.001	1.066 [1.016–1.119]	0.01
Education level				
College level ≤50%	Ref.		Ref.	
College level >50%	0.929 [0.883–0.979]	0.006	0.926 [0.870–0.986]	0.017
Median household income				
≤USD 50,000	Ref.		Ref.	
USD 50,000–100,000	1.025 [0.991–1.059]	0.151	0.999 [0.966–1.033]	0.955
>USD 100,000	0.975 [0.908–1.046]	0.473	0.963 [0.885–1.048]	0.383

Abbreviations: HR, hazard ratio; CI, confidence interval; AIAN, American Indian/Alaska Native; API, Asian or Pacific Islander; Tcc, transitional cell carcinoma; Scc, squamous cell carcinoma; Nec, neuroendocrine carcinoma; Ac, adenocarcinoma; Oet, other epithelial tumors; TURBT, transurethral resection of bladder tumor; PC, partial cystectomy; RC, radical cystectomy. * A two-sided *p*-value < 0.05 was considered statistically significant.

## Data Availability

Publicly available datasets were analyzed in this study. These data can be found in the SEER database (https://seer.cancer.gov/, accessed on 22 November 2021).

## Abbreviations

BC: bladder cancer; CVM: cardiovascular mortality; SMR: standardized mortality ratio; CVD: cardiovascular disease; 95% CI: 95% confidence interval; SEER: Surveillance, Epidemiology, and End Results; CIF: cumulative incidence function; API: Asian or Pacific Islander; AIAN: American Indian/Alaska Native; Tcc: transitional cell carcinoma; Scc: squamous cell carcinoma; Ac: adenocarcinoma; Nec: neuroendocrine carcinoma; Oet: other epithelial tumors; TURBT: transurethral resection of bladder tumor; PC: partial cystectomy; RC: radical cystectomy.
